# Multi-omics identification of novel biomarkers and therapeutic targets for Parkinson's disease: from transcriptome to drug interaction

**DOI:** 10.3389/fnagi.2026.1870056

**Published:** 2026-09-22

**Authors:** Bingbing Zhao, Zhe Shi, Xiuming Pang, Yue Qi

**Affiliations:** 1 Second Affiliated Hospital of Heilongjiang University of Chinese Medicine, Harbin, China; 2 Heilongjiang Sengong General Hospital, Harbin, China; 3 College of Bioinformatics Science and Technology, Harbin Medical University, Harbin, China

**Keywords:** acupuncture, biomarkers, machine learning, Parkinson, transcriptomic analysis

## Abstract

**Introduction:**

Parkinson's disease (PD) is a progressive neurodegenerative disorder with limited therapeutic options. Acupuncture may serve as a complementary intervention, but its molecular mechanisms remain unclear. This study integrated bulk and single-cell transcriptomic datasets to identify PD-associated diagnostic candidate genes and explore their relationship with acupuncture-related temporal expression patterns.

**Methods:**

PD-associated differentially expressed genes were identified from GSE68719. Time-series analysis of GSE178470 characterized ascending or descending expression trajectories in longitudinal blood transcriptomes from one patient with PD sampled at baseline and after 5 and 8 acupuncture sessions. Genes showing directionally opposite patterns between PD-associated dysregulation and post-acupuncture temporal changes were intersected, followed by feature selection using LASSO, Random Forest, Boruta, and XGBoost. Diagnostic performance was evaluated in GSE68719. Single-cell transcriptomic analysis, molecular docking, and MPP+-treated SH-SY5Y cells were used for further exploratory characterization and validation.

**Results:**

Eleven candidate genes were identified, of which four were prioritized by machine-learning approaches. The four-gene panel discriminated patients with PD from healthy controls in GSE68719, with an apparent AUC of 0.873, sensitivity of 0.690, specificity of 0.864, accuracy of 0.795, precision of 0.769, F1 score of 0.727, and Brier score of 0.134. These metrics reflect PD-versus-control classification rather than prediction of acupuncture response. Single-cell analysis mapped cell type-specific expression across human midbrain populations. Molecular docking provided exploratory predicted binding poses. BAG3 and HSPB1 were upregulated in MPP+-treated SH-SY5Y cells.

**Discussion:**

These findings provide an integrative framework for prioritizing PD-associated candidate genes with exploratory acupuncture-related temporal patterns. Further validation in larger longitudinal cohorts and functional studies is required.

## Introduction

Parkinson's disease (PD) is a common and progressive neurodegenerative disorder characterized by the selective loss of dopaminergic neurons in the substantia nigra pars compacta and the presence of Lewy bodies (Eriksen et al., 2005). As the global population ages, the incidence and societal burden of PD continue to rise. Although dopamine replacement therapies, such as levodopa and its derivatives, remain the mainstay of symptomatic treatment, they are often associated with long-term complications including motor fluctuations and dyskinesias. Moreover, these treatments do not halt the underlying neurodegenerative process (Paolini Paoletti et al., 2019; Tolosa et al., 2021; Salat and Tolosa, 2013). Therefore, there is an urgent need for therapeutic strategies that are not only effective but also safe and capable of modifying disease progression.

Acupuncture, a core modality of traditional Chinese medicine (TCM), has been increasingly studied in the context of neurological disorders, including PD (Chen et al., 2015). Clinical and experimental studies have suggested that acupuncture may alleviate motor symptoms, improve mood and sleep disturbances, and potentially slow disease progression in PD patients (Xia et al., 2023). Mechanistic investigations indicate that acupuncture may exert its neuroprotective effects through the regulation of neurotransmitter levels, reduction of neuroinflammation, and enhancement of mitochondrial function (Chen et al., 2024). However, most of these studies have focused on macroscopic or localized effects, and there is a lack of comprehensive molecular evidence—particularly at the transcriptomic level—demonstrating how acupuncture alters gene expression profiles relevant to PD pathogenesis (Choi et al., 2011; Yeo et al., 2013, 2015).

In parallel, advances in high-throughput sequencing technologies and computational biology have enabled the systematic investigation of gene expression changes in complex diseases. Transcriptomic analyses, including bulk RNA sequencing and single-cell RNA sequencing (scRNA-seq), have been widely applied to uncover key molecular pathways and therapeutic targets in PD (Wang et al., 2024). Despite this, few studies have utilized these approaches to investigate the molecular responses to acupuncture treatment. Moreover, machine-learning approaches have increasingly been used to prioritize diagnostic or disease-associated gene panels in PD, but their application to acupuncture-related transcriptomic hypotheses remains limited (Zhao et al., 2021; Mei et al., 2021; Shokrpour et al., 2025). This represents a significant gap, particularly in efforts to establish robust biomarkers for treatment responsiveness and to understand how acupuncture may modulate the immune microenvironment in PD.

In this study, we aimed to address these gaps by constructing an integrative transcriptomic framework to identify PD-associated candidate genes and explore their relationship with acupuncture-related temporal expression patterns. We first performed time-course transcriptomic analysis using the Mfuzz algorithm to describe dynamic gene-expression trajectories in longitudinal blood samples from one representative patient with PD during acupuncture treatment. By integrating an independent PD case-control dataset, we further narrowed these candidates to genes that were dysregulated in PD and showed directionally opposite temporal patterns after acupuncture, highlighting them as hypotheses for future validation rather than confirmed markers of disease modification.

To prioritize PD-associated diagnostic candidate genes, we employed four complementary machine learning approaches—Random Forest, LASSO regression, XGBoost, and Boruta feature selection. Importantly, these genes showed discriminatory ability for distinguishing PD patients from healthy controls, indicating their potential utility as PD-associated diagnostic candidates rather than validated markers of acupuncture treatment response.

To unravel the functional relevance of these core genes, we conducted a series of in *silico* and *in vitro* analyses. Computationally, immune cell infiltration profiling linked these genes to components known to participate in PD pathophysiology, while single-cell RNA sequencing mapped their expression specifically to microglia, endothelial cells, and ependymal cells. Moreover, molecular docking provided exploratory in *silico* predictions of possible protein-ligand binding poses with established dopaminergic drugs. Crucially, to validate the intrinsic pathological involvement of these computationally derived targets, we performed *in vitro* experiments using a PD cell model. This biological validation showed stress-induced upregulation of BAG3 and HSPB1 in an MPP+-treated cellular model, supporting their stress-responsive expression rather than proving a causal role in acupuncture-induced neuroprotection.

Collectively, our integrated computational and experimental framework prioritizes PD-associated candidate genes with exploratory acupuncture-related temporal patterns. This study generates testable hypotheses for future studies designed to evaluate whether baseline or longitudinal gene-expression changes are associated with clinical acupuncture outcomes in PD.

## Materials and methods

### Data sources

Publicly available transcriptomic datasets were retrieved from the Gene Expression Omnibus (GEO) database. Specifically, according to the original GEO description, GSE178470 includes longitudinal blood transcriptomic profiles from one representative patient with PD undergoing acupuncture treatment, with RNA-seq samples collected at baseline (pre-treatment), 5 sessions, and 8 sessions post-treatment. The GSE68719 dataset includes differential gene expression profiles comparing PD patients to healthy controls. To validate the robustness and generalizability of our predictive model, an independent cohort from GSE239454 was employed. Additionally, single-cell RNA sequencing data from human midbrain (GSE157783) were used to examine the expression patterns of core feature genes across distinct neural cell types. Cell type annotations in GSE157783 were adopted from the original study to ensure consistency (Smajić et al., 2022). All datasets were downloaded and processed according to the original study protocols.

### Cell culture and treatment

The human neuroblastoma SH-SY5Y cell line present in this study was obtained from the National Collection of Authenticated Cell Cultures (Shanghai, China). The cells were cultured in Dulbecco's Modified Eagle Medium (DMEM) supplemented with 10% fetal bovine serum (FBS) and 1% penicillin-streptomycin at 37 °C in a humidified incubator containing 5% CO_2_. To establish the *in vitro* PD model, SH-SY5Y cells were treated with 1-methyl-4-phenylpyridinium (MPP+) at a concentration of 0.5 mM for 24 h. Control cells (vehicle group) were treated with an equivalent volume of the corresponding vehicle.

### RT-qPCR

Following RNA extraction with TransZol (TransGen, Beijing, China) and resuspension in DEPC water, RNA concentrations were evaluated using a NanoDrop 2000 spectrophotometer (Thermo Fisher, USA). cDNA was synthesized from 1 μg of total RNA using the 5 × TransScript All-in-One SuperMix and gDNA Remover in a 20 μL reaction volume (incubated at 50 °C for 5 min, followed by 85 °C for 2 min). Subsequent qPCR analysis was performed on a Roche LightCycler 96 system utilizing the 2 × TransStart Top Green qPCR SuperMix (TransGen). The 20 μL PCR mixtures—containing 1 μL of cDNA, 0.4 μL each of 10 μM forward and reverse primers, and 8.2 μL of RNase-free water—underwent 45 amplification cycles (94 °C for 5 s, 58 °C for 15 s, and 72 °C for 1 min). All primers (Table 1), including those targeting *HSPB1, BAG3*, and *GAPDH*, were procured from Genewiz (Suzhou, China). Relative gene expression fold changes were calculated via the comparative 2∧-ΔΔCt method, normalized to *GAPDH*.

**Table 1:** The primer sequences.

Primer	Sequence (5^′^-3^′^)
HSPB1 forward (homo)	AGTTTCCTCCTCCCTGTC
HSPB1 reverse (homo)	TCATCGGATTTTGCAGCTT
BAG3 forward (homo)	ATGACCCATCGAGAAACTG
BAG3 reverse (homo)	TCTTTGCGGATCACTTGAAT
GAPDH forward (homo)	GGTGGTCTCCTCTGACTTCAACA
GAPDH reverse (homo)	GTTGCTGTAGCCAAATTCGTTGT

### Western blotting

For protein extraction, cells seeded in 6- or 12-well plates were grown to 80% confluence. Cell lysis was achieved by adding 100 μL of RIPA buffer (Beyotime, China) supplemented with 1% PMSF, followed by a 20-min incubation on ice. The lysates underwent centrifugation at 12,000 × g for 20 min at 4 °C to isolate the protein-rich supernatant. A BCA Protein Assay Kit (Beyotime) was utilized for protein quantification. Equal amounts of extracted proteins were separated by 10% SDS-PAGE and transferred onto PVDF membranes (Millipore, USA). The membranes were subjected to a 1-h blocking step with skimmed milk at room temperature, followed by overnight incubation at 4 °C with primary antibodies against GAPDH (1:1000), BAG3 (1:1000), and HSPB1 (1:1000) (all purchased from Proteintech, Wuhan, China). Following a triple wash sequence with 1 × TBST, the membranes were incubated for 1 h at room temperature with horseradish peroxidase (HRP)-conjugated secondary antibodies (goat anti-rabbit or goat anti-mouse IgG, 1:5000; Proteintech). Visualization of the target protein bands was executed on a Tanon 5200 Chemi-Image System (Biotanon, China), and densitometric analysis was conducted using ImageJ software.

### Machine learning analysis

To prioritize PD-associated diagnostic candidate genes from the 11 intersected candidates, this study integrated four complementary machine-learning approaches: Least Absolute Shrinkage and Selection Operator (LASSO) regression, Random Forest (RF), Boruta feature selection, and Extreme Gradient Boosting (XGBoost). Differentially expressed candidate genes were first used as input features to assess and select important variables. Briefly, LASSO regression employs an L1 regularization penalty to eliminate redundant variables; Random Forest evaluates variable importance via an ensemble of decision trees; Boruta performs stable feature selection by comparing real features against randomized shadow features; and XGBoost utilizes a gradient boosting approach. To enhance selection robustness, genes supported by complementary algorithms and retained by LASSO were used to define the core gene set. Finally, diagnostic classification models were constructed based on this locked core gene set to distinguish PD patients from healthy controls. Feature screening was performed before model evaluation and was not nested within each cross-validation fold; therefore, cross-validation results were interpreted as conditional performance estimates of the locked gene panel rather than as fully unbiased estimates of the entire discovery pipeline. SHAP analysis was applied to the trained XGBoost model to interpret the contribution of each candidate gene to model predictions and was not used as an independent feature-selection algorithm. Model performance was reported using AUC with 95% confidence intervals when available, sensitivity, specificity, accuracy, precision, F1 score, Brier score, and the classification threshold; unless otherwise specified, the classification threshold was 0.5. The model's generalizability was rigorously validated via 5-fold cross-validation and tested on an independent external dataset.

### Molecular docking analysis

The 3D structures of the drugs were obtained from the PubChem database, and the spatial structures of the target proteins were downloaded from the Protein Data Bank (PDB). Molecular docking simulations were performed using AutoDock Vina software to generate exploratory protein-ligand binding poses and predicted binding energies. Finally, PyMOL software was employed to visualize the docking results and interaction patterns between proteins and ligands.

### Bioinformatics and statistical analysis

Differential expression analysis was performed using the limma R package, with thresholds set at |log_2_ fold change| > 1 and adjusted *P* value < 0.05. Temporal expression pattern clustering during acupuncture intervention was conducted using Mfuzz to describe genes showing ascending or descending expression trajectories across the three time points from the representative patient. Because the RNA-seq time course lacked biological replication, these clusters were not used for population-level statistical inference. Functional enrichment analyses, including Gene Ontology (GO), Kyoto Encyclopedia of Genes and Genomes (KEGG), and Gene Set Enrichment Analysis (GSEA), were carried out using the cluster Profiler R package. Gene sets were sourced from the Molecular Signatures Database (MSigDB).

## Results

### Exploratory temporal expression patterns during acupuncture treatment in one gait-responsive patient with PD

To describe temporal gene-expression patterns during acupuncture intervention, we applied the Mfuzz clustering algorithm to the longitudinal blood transcriptomic profiles of one representative patient with PD from the GSE178470 dataset, sampled at baseline, 5 sessions of acupuncture, and 8 sessions of acupuncture. This descriptive analysis identified six temporal expression patterns, among which four clusters showed sustained ascending or descending trajectories across the three sampled time points (Figure 1A). Specifically, a total of 7,878 genes showed a progressive increase in expression (Clusters 3 and 4), whereas 3,631 genes displayed a continuous decline (Clusters 1 and 2).

**Figure 1:**
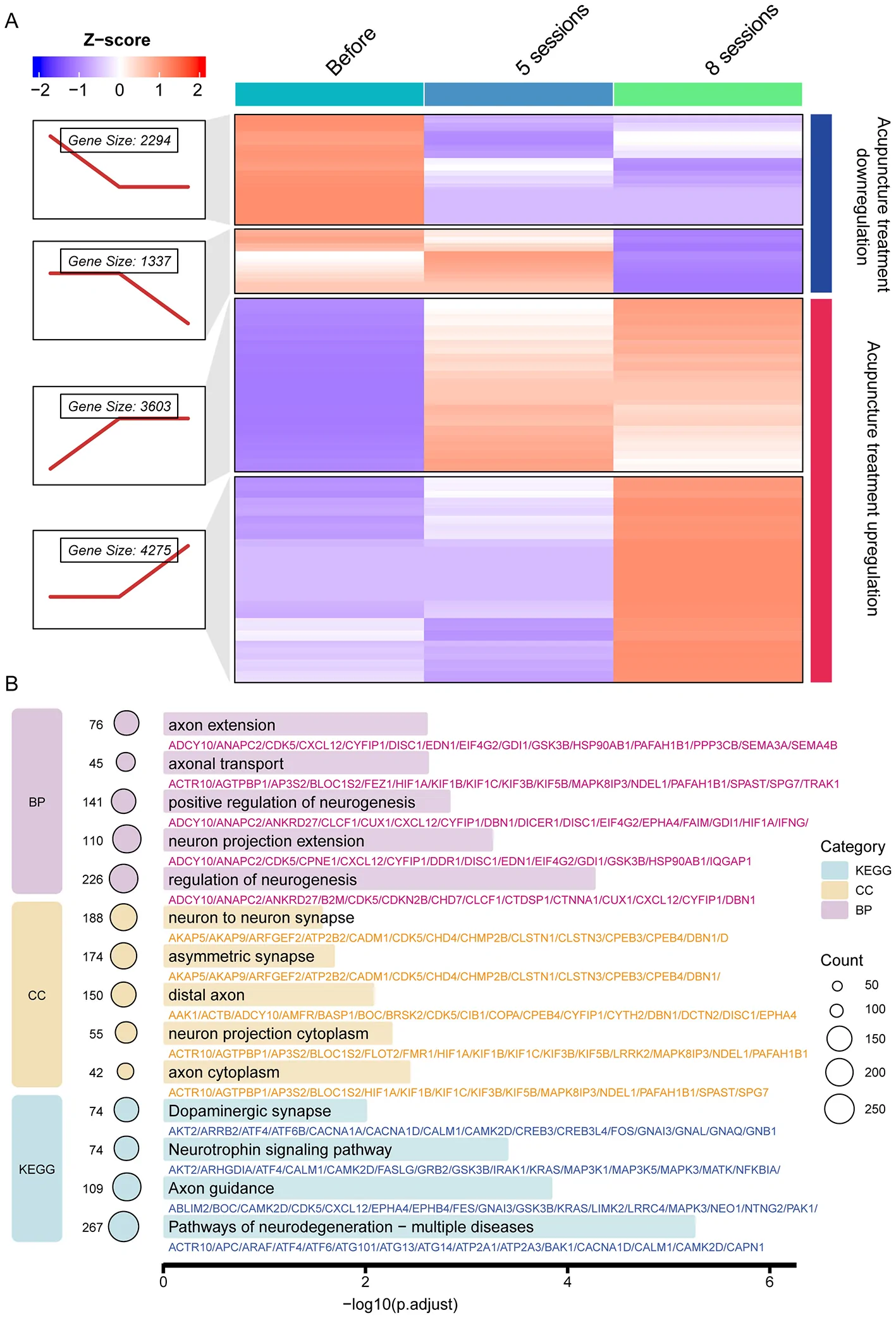
Exploratory time-series clustering and functional enrichment analysis of transcriptomic changes during acupuncture treatment in one representative patient with Parkinson's disease (PD). **(A)** Mfuzz clustering analysis of the GSE178470 RNA-seq time course from one representative patient with PD identified six descriptive gene-expression clusters across treatment stages (0, 5, and 8 sessions of acupuncture). **(B)** Functional enrichment analysis (clusterProfiler, FDR < 0.05) of dynamically expressed genes revealed significant enrichment in nervous system-related pathways.

Functional enrichment analysis of genes showing temporal expression patterns revealed enrichment in biological processes and pathways related to nervous system homeostasis (Figure 1B). Crucially, KEGG analysis highlighted core PD-associated pathways, most notably the “Dopaminergic synapse” and “Pathways of neurodegeneration—multiple diseases” (FDR < 0.05). Furthermore, GO terms were significantly enriched in synaptic function and plasticity, including “regulation of neurogenesis” and “neuron-to-neuron synapse.” Together, these findings suggest that genes showing temporal changes in this representative patient were enriched in neurobiological pathways relevant to PD; however, because the RNA-seq time course lacked biological replication, these results should be interpreted as hypothesis-generating rather than as evidence of population-level acupuncture-induced neuroprotection.

### Identification of PD-associated diagnostic candidate genes using multiple machine-learning algorithms

To identify PD-associated candidate genes, we performed differential gene expression analysis using the GSE68719 dataset comparing PD patients with healthy controls. This analysis revealed 49 upregulated and 17 downregulated genes in the PD group. Intersecting these PD-associated differentially expressed genes with the descriptive acupuncture-related temporal gene set from GSE178470 yielded 11 candidate genes (Figure 2B). These genes showed directionally opposite patterns between PD status and the post-acupuncture time course, but this overlap should be interpreted as exploratory rather than as proof of therapeutic reversal. Among these 11 candidates, eight genes (*GPCPD1, NAP1L5, SVOP, MAL2, CRYM, SCN2A, SLC4A10*, and *NCALD*) were lower in PD and increased over the acupuncture time course, whereas three genes (HSPB1, KCNE4, and BAG3) were higher in PD and decreased over the acupuncture time course. Because the time-course RNA-seq data came from one representative patient, these reversal-like patterns were considered descriptive.

**Figure 2:**
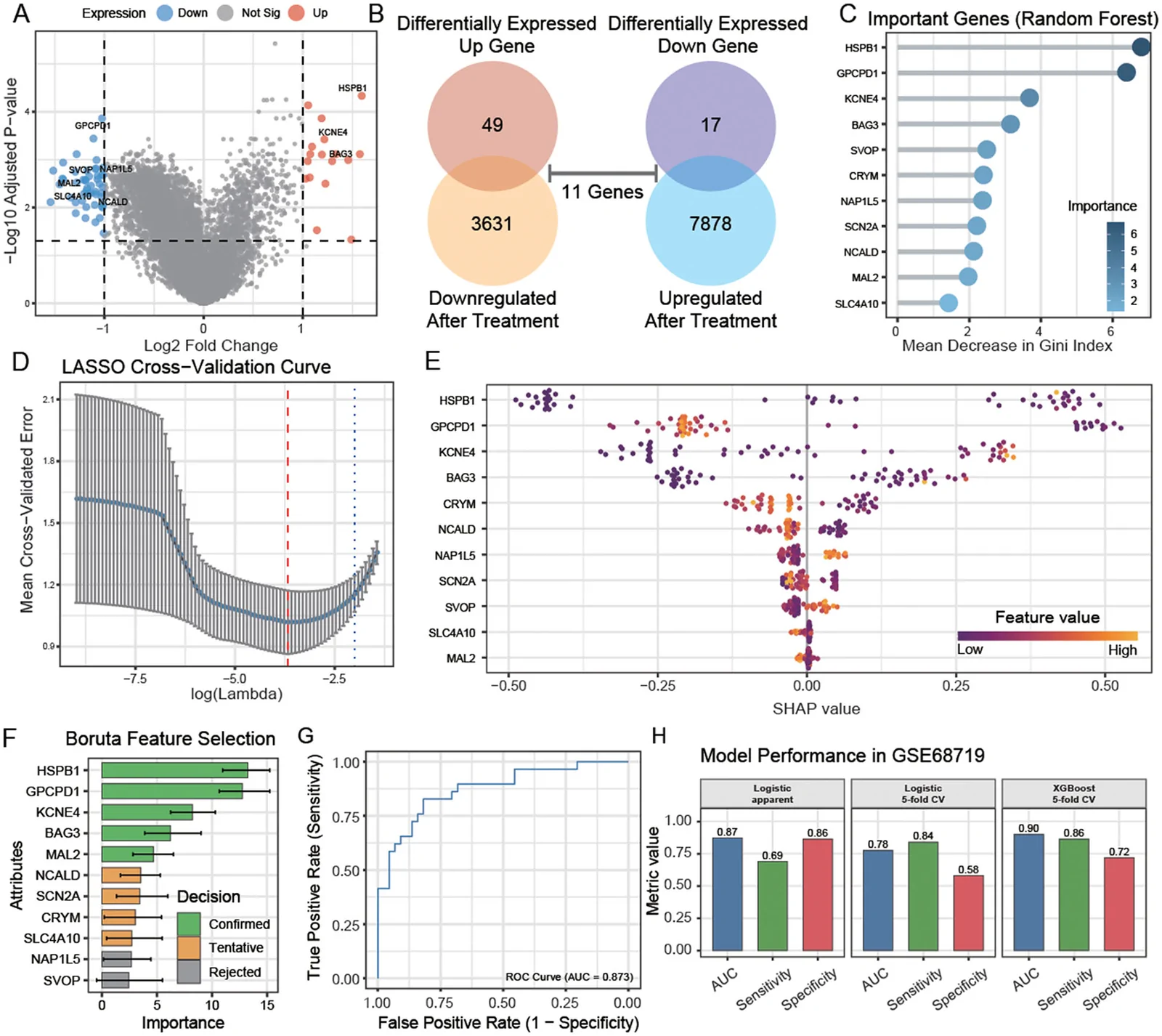
Identification and evaluation of PD-associated diagnostic candidate genes. **(A)** Volcano plot showing differentially expressed genes (DEGs) between PD patients and healthy controls in GSE68719. Blue dots indicate downregulated genes, red dots indicate upregulated genes, and gray dots indicate non-significant genes. **(B)** Intersection of PD-associated DEGs from GSE68719 with genes showing exploratory temporal expression patterns after acupuncture in GSE178470, yielding 11 candidate genes. **(C)** Random Forest ranking of candidate-gene importance based on mean decrease in Gini index. **(D)** LASSO cross-validation curve for candidate-gene selection. **(E)** SHAP summary plot showing feature contributions in the XGBoost model. **(F)** Boruta feature-selection results for the 11 candidate genes. **(G)** ROC curve of the four-gene diagnostic model in GSE68719. **(H)** Model- performance summary in GSE68719, including apparent logistic-model performance and 5-fold cross-validation performance of logistic and XGBoost models.

To rigorously assess feature importance and select the most robust targets, we employed four complementary machine learning algorithms: Random Forest, XGBoost, Boruta, and Least Absolute Shrinkage and Selection Operator (LASSO) regression. Random Forest and XGBoost identified *HSPB1* as the top-ranking gene, followed by *GPCPD1*. Similarly, the Boruta algorithm ranked *GPCPD1* highest, followed by *HSPB1*. Concurrently, LASSO regression retained *GPCPD1, HSPB1, KCNE4*, and *BAG3* as key non-zero features (Figures 2C–F).

Integrating the results of the four feature-prioritization approaches, we established a four-gene diagnostic candidate signature (*GPCPD1, HSPB1, KCNE4*, and *BAG3*). Based on this signature, a Random Forest classification model was constructed. To evaluate the model's diagnostic performance and generalizability, we performed Receiver Operating Characteristic (ROC) curve analysis. In GSE68719, the four-gene logistic model showed an apparent AUC of 0.873 (95% CI, 0.789–0.958), with sensitivity of 0.690, specificity of 0.864, accuracy of 0.795, precision of 0.769, F1 score of 0.727, and Brier score of 0.134 at a threshold of 0.5. Five-fold cross-validation yielded an AUC of 0.776 for the logistic model and 0.900 for the XGBoost model (Figures 2G, H). These metrics reflect PD vs. healthy-control diagnostic discrimination.

Collectively, this multi-algorithm strategy effectively mitigates the bias inherent to any single method. The identified four-gene signature provides a candidate diagnostic framework for PD and a hypothesis-generating basis for future replicated studies of acupuncture-related transcriptomic changes.

### Pathway associations and single-cell expression patterns of the four-gene model

In the GSE68719 dataset, the four core genes (*GPCPD1, HSPB1, KCNE4, BAG3*) were significantly differentially expressed between PD patients and healthy controls (*P* < 0.05, Figure 3A), reinforcing their potential involvement in PD pathogenesis. To further explore their functional implications, we analyzed the correlation between these genes and critical biological pathways. Using single-sample gene set enrichment analysis (ssGSEA), we constructed functional gene sets covering autophagy, oxidative stress, dopamine signaling, and synaptic signaling. The results demonstrated that the four genes exhibited moderate-to-strong correlations with oxidative stress (correlation coefficient: 0.58–0.65), autophagy (0.40–0.73), and synaptic signaling (0.57–0.65), but much weaker correlations with dopamine signaling (0.01–0.16) (Figure 3A). These findings suggest that the four candidate genes are associated with pathway activity scores related to oxidative stress, autophagy, and synaptic function, all of which are relevant to neurodegenerative processes; because these analyses are correlation-based, they do not establish causal regulation of these pathways.

**Figure 3:**
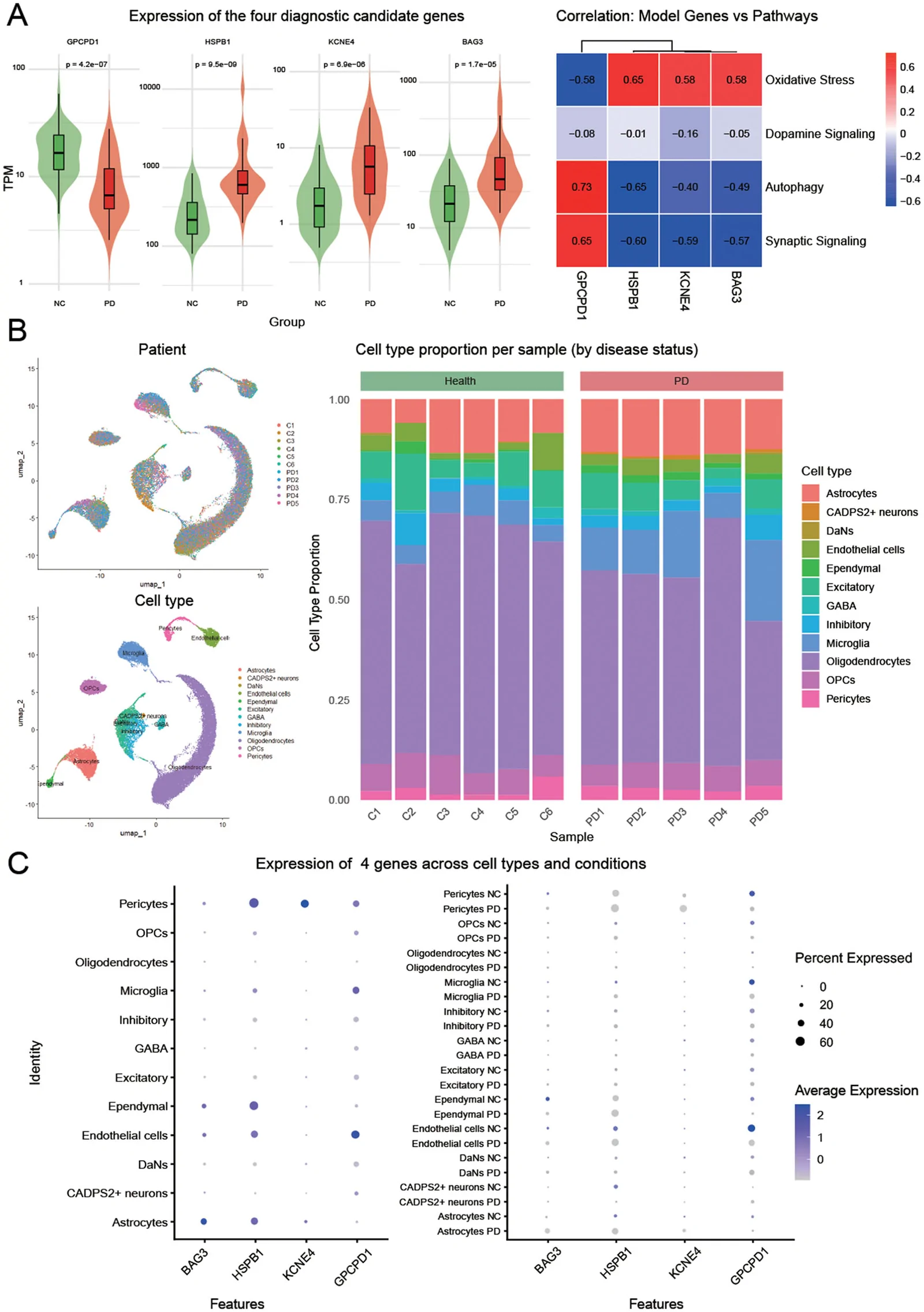
Expression patterns and functional implications of four core genes in Parkinson's disease (PD). **(A)** Violin plots showing expression of the four core genes (GPCPD1, HSPB1, KCNE4, BAG3) between PD patients and healthy controls in the GSE68719 dataset, together with pathway correlation heatmaps. Heatmap shows correlations between these genes and key biological pathways (oxidative stress, autophagy, dopamine signaling, and synaptic signaling), with strongest associations observed for oxidative stress, autophagy, and synaptic signaling. **(B)** Single-cell RNA-seq analysis from the GSE157783 dataset. UMAP plots display clustering by patient and cell type after Harmony batch correction. Bar plot shows altered proportions of cell types between PD and controls, with notable differences in ependymal cells and microglia. **(C)** Dot plot of gene expression across cell types and conditions. *GPCPD1* was more highly expressed in microglia, ependymal, and endothelial cells in controls compared with PD patients, highlighting its potential role in maintaining cellular homeostasis and neuronal support.

Notably, oxidative stress and autophagy are two major pathological hallmarks of PD: the former exacerbates neuronal damage, while the latter is crucial for neuronal survival and protein clearance (Dias et al., 2013; Cerri and Blandini, 2019). The strong correlation with synaptic signaling further supports the potential role of these genes in neuronal communication and plasticity. In contrast, their weak association with dopamine signaling indicates that they are unlikely to be direct regulators of dopamine metabolism or transmission, but may instead affect PD phenotypes indirectly by modulating upstream or parallel neurobiological processes.

To delineate the cell-type-specific expression profiles of these targets, we analyzed the GSE157783 single-cell transcriptomic dataset. Following Harmony-based batch correction and Uniform Manifold Approximation and Projection (UMAP) dimension reduction, we mapped the core genes across distinct neural cell populations. At the donor/sample level, we observed descriptive differences in the proportions of ependymal cells and microglia between PD and healthy-control samples (Figure 3B). This pattern is consistent with reported glial involvement in PD, although the present single-cell analysis should be interpreted descriptively and does not treat individual cells as independent biological replicates. In particular, GPCPD1 showed higher expression levels and a greater proportion of positive cells in microglia, ependymal, and endothelial cells in healthy controls compared with PD patients (Figure 3C). This pattern suggests that GPCPD1 may be related to cellular homeostasis and neuro-glial support.

Collectively, the pathway correlation analyses and single-cell transcriptomic profiling provide functional and cellular context for the four-gene panel in PD. By mapping these genes to specific glial and endothelial populations, our results generate hypotheses for future cell-type-specific validation and for investigating whether acupuncture-related temporal changes are connected to these cellular contexts.

### Exploratory molecular docking analysis of core gene-encoded proteins with anti-Parkinsonian drugs

To explore possible in *silico* binding poses between the identified candidate proteins and clinically used antiparkinsonian drugs, we selected three representative dopamine precursors–Levodopa, Melevodopa, and Etilevodopa–for molecular docking analysis with GPCPD1, KCNE4, BAG3, and HSPB1 (Poewe et al., 2017; Oertel and Schulz, 2016). The results demonstrated that GPCPD1 exhibited the lowest binding energies with all three drugs (−7.1, −7.1, and −6.9 kcal/mol, respectively), suggesting relatively favorable predicted binding poses among the candidates. In contrast, BAG3 displayed weaker affinities (−4.4 to −4.8 kcal/mol), while KCNE4 showed moderate binding with Melevodopa and Etilevodopa (−4.6 and −4.7 kcal/mol). HSPB1 demonstrated a relatively strong binding affinity solely with Etilevodopa (−6.1 kcal/mol) (Figure 4). These findings suggest that the candidate proteins may differ in their predicted docking profiles with dopaminergic drugs, with GPCPD1 showing relatively lower predicted binding energies among the tested proteins. Together with its disease-associated expression pattern and exploratory acupuncture-related temporal change, this result provides a structural hypothesis that may help prioritize GPCPD1 for future validation. However, these docking results should be interpreted as preliminary in *silico* evidence and require confirmation by physical binding assays and functional experiments.

**Figure 4:**
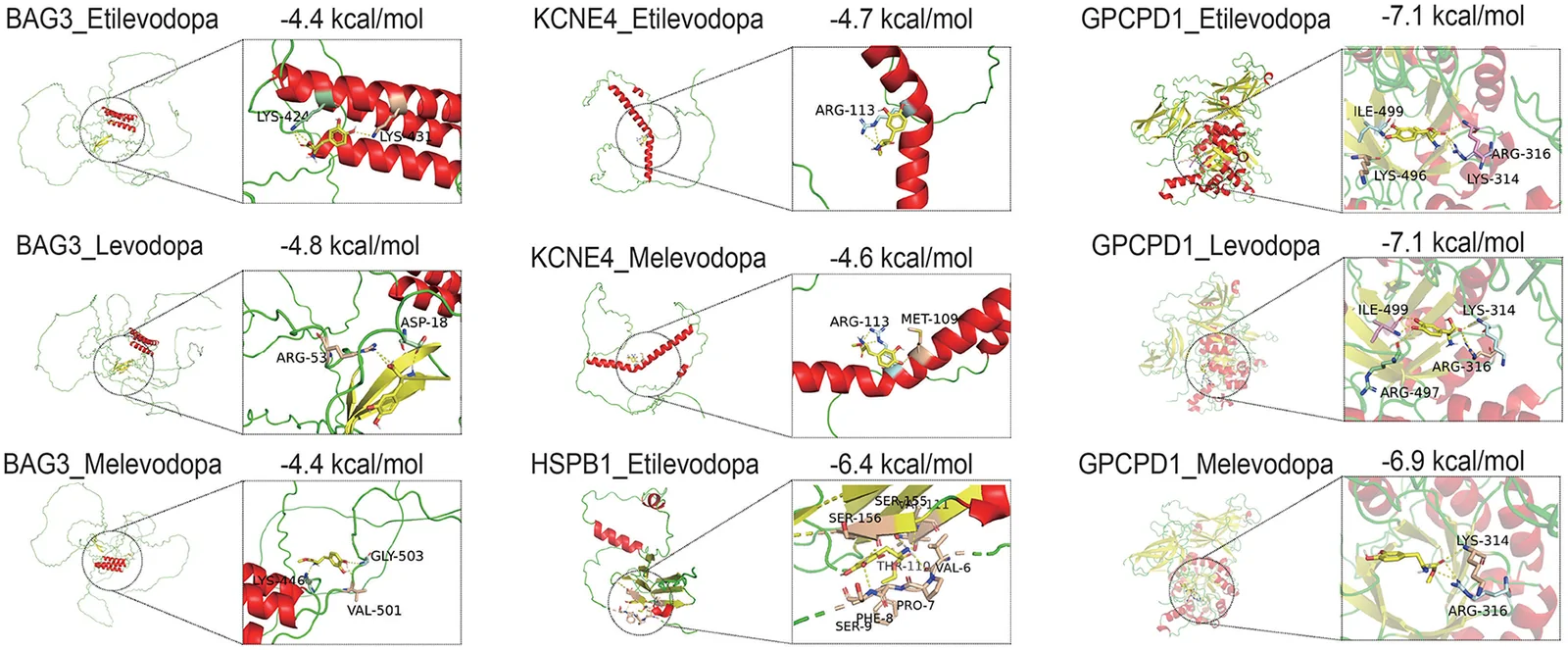
Molecular docking analysis of core gene-encoded proteins with dopaminergic drugs.

### BAG3 and HSPB1 are upregulated in an MPP+-induced cellular stress model

Because BAG3 and HSPB1 were included in the prioritized four-gene panel, we examined whether their expression changed in an MPP+-induced cellular stress model. Therefore, we established an *in vitro* PD model using MPP+-induced SH-SY5Y cells to investigate their basal stress responses. Both the mRNA and protein levels of BAG3 and HSPB1 were upregulated in the MPP+-treated group compared with the vehicle control group (Figure 5). Specifically, the relative mRNA expression of *BAG3* (*P* = 0.0004) and *HSPB1* (*P* = 0.0198) showed a marked increase following neurotoxicity (Figures 5D, E). Similarly, densitometric analysis of the immunoblots confirmed that the protein levels of BAG3 (*P* = 0.0458) and HSPB1 (*P* = 0.0286) were significantly elevated in response to the PD-like insult (Figures 5A–C). These results indicate that BAG3 and HSPB1 respond to MPP+-induced cellular stress.

**Figure 5:**
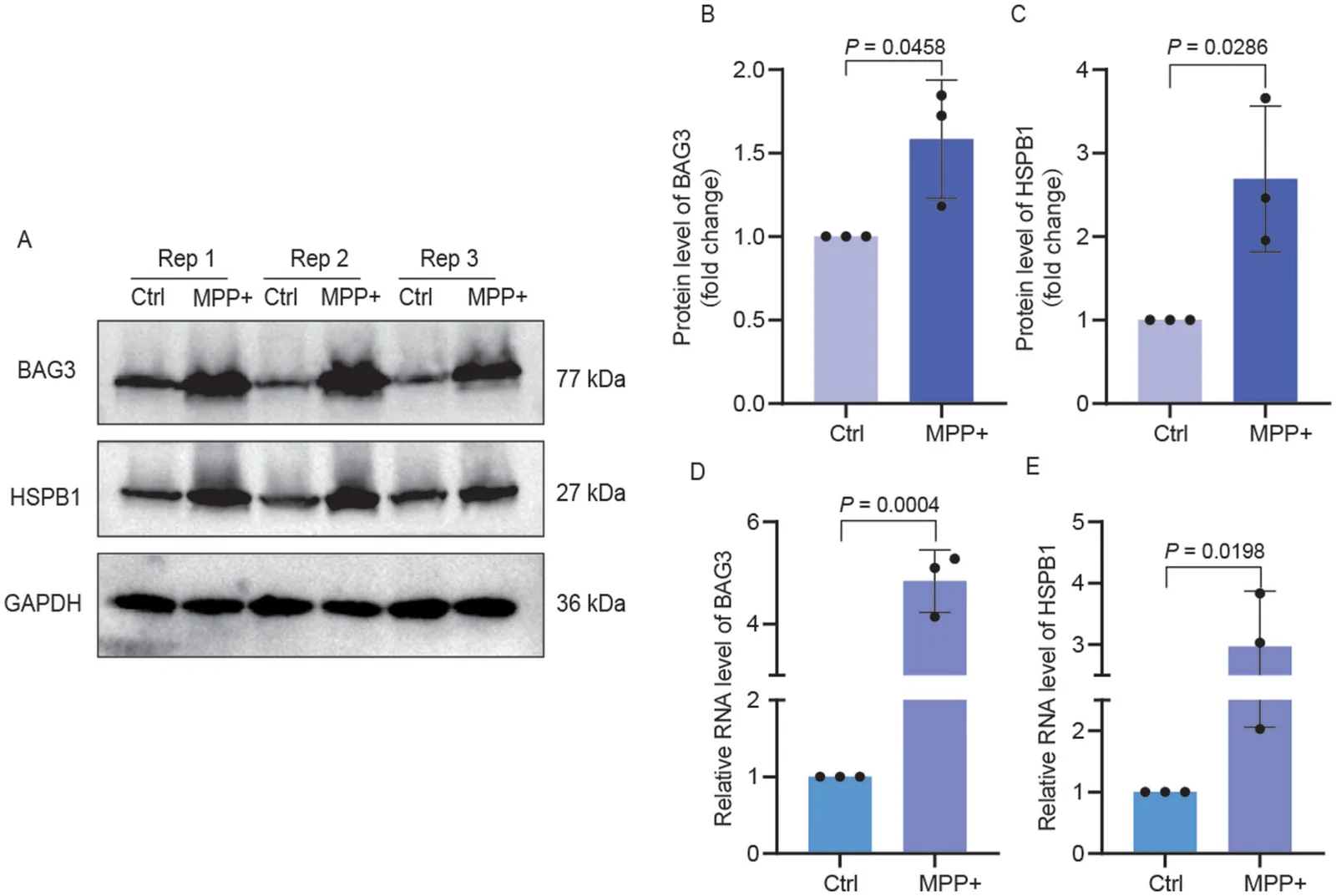
BAG3 and HSPB1 expression in an MPP+-induced cellular stress model. **(A)** Representative western blot images showing BAG3 and HSPB1 protein expression in control cells (Ctrl) and MPP+-treated SH-SY5Y cells. GAPDH was used as the loading control, and Rep 1–3 indicate three independent biological replicates. **(B, C)** Quantification of BAG3 and HSPB1 protein levels normalized to GAPDH. **(D, E)** RT-qPCR analysis of BAG3 and HSPB1 mRNA levels in MPP+-treated cells compared with control cells. Data are presented as mean ± SD. Statistical comparisons were performed using two-sided unpaired Student's *t*-tests. These results indicate that BAG3 and HSPB1 respond to MPP+-induced cellular stress.

## Discussion

Parkinson's disease (PD), a common neurodegenerative disorder, poses a significant challenge in neuroscience due to its complex pathogenesis and limited therapeutic options (Jankovic and Tan, 2020). Acupuncture, an essential component of traditional Chinese medicine, has recently been shown to alleviate PD symptoms and improve patient quality of life (Li et al., 2025; Guo et al., 2024); however, its underlying molecular mechanisms remain largely unexplored. Currently, molecular biomarkers associated with acupuncture intervention are poorly defined, hindering its clinical translation and the development of personalized therapeutic strategies. Therefore, identifying PD-associated candidate genes and exploring their relationship with acupuncture-related transcriptomic changes may provide useful molecular hypotheses for future mechanistic and clinical studies.

In this study, we prioritized PD-associated diagnostic candidate genes by integrating public bulk and single-cell transcriptomic datasets with descriptive time-series analysis and complementary machine-learning approaches. We applied the Mfuzz algorithm to cluster the time-series transcriptomic data from one representative patient with PD at 0, 5, and 8 sessions of acupuncture treatment from the GSE178470 dataset. Mfuzz is particularly suitable for time-series data due to its robustness to noise and ability to identify gene clusters with similar dynamic expression patterns over time. Considering that acupuncture exerts gradual, time-dependent molecular effects, screening gene clusters that exhibited sustained ascending or descending trajectories (four clusters comprising 7,878 gradually increasing and 3,631 continuously decreasing genes) represents a critical and necessary first step. This approach identified genes with exploratory acupuncture-related temporal patterns, laying the foundation for subsequent intersection with PD-associated targets. Functional enrichment analysis revealed that these genes showing temporal expression patterns were enriched in pathways related to neurogenesis regulation, neuron-to-neuron synapse formation, and multiple neurodegenerative disease pathways, suggesting their potential relevance to PD-related neurobiological processes. Our application of Mfuzz therefore provides a hypothesis-generating view of temporal expression changes during acupuncture. To prioritize the most robust candidates, we employed a complementary machine learning strategy integrating four algorithms—LASSO, Random Forest, Boruta, and XGBoost. Feature importance assessment and cross-validation across these models enabled the robust selection of a core 4-gene signature (*GPCPD1, HSPB1, KCNE4, BAG3*). This multi-algorithm approach effectively mitigates the bias inherent to any single model, enhancing both the stability and generalizability of the selected gene set.

Importantly, the identification of a consistent gene panel across diverse computational frameworks suggests that these genes are not merely artifacts of statistical modeling but reflect genuine biological nodes. In particular, *HSPB1* (Chung et al., 2016) and *BAG3* (Ying et al., 2022) have been previously implicated in neuroprotection and protein homeostasis, lending biological plausibility to their roles in PD and acupuncture-related temporal expression changes. Meanwhile, *GPCPD1* and *KCNE4*, though less studied in the context of PD, emerged from our analyses and warrant further biological validation.

Furthermore, single-cell transcriptomic analysis (GSE157783) provided cell-type-specific resolution to corroborate the bulk RNA-seq findings. After batch correction with Harmony and maintaining the original cell-type annotations, donor-level cell-type composition suggested differences in ependymal cells and microglia between PD and healthy-control samples. Notably, *GPCPD1* exhibited higher expression levels in microglia, ependymal, and endothelial cells of healthy controls compared to PD samples. This suggests a potential role for *GPCPD1* in maintaining cellular homeostasis or exerting protective effects in these specific cellular niches. The integration of single-cell data not only contextualizes population-level differences but also maps specific gene expression to distinct neuro-glial populations, providing a clear rationale for future cell-specific functional experiments.

In *silico* molecular docking analysis showed that the GPCPD1 protein had relatively low predicted binding energies with three dopamine precursor drugs (Levodopa, Melevodopa, and Etilevodopa; approximately −6.9 to −7.1 kcal/mol), suggesting favorable predicted docking poses in this exploratory analysis. In contrast, BAG3 showed weaker interactions with these drugs, while KCNE4 demonstrated moderate affinity. Notably, Levodopa-based drugs are standard clinical treatments for PD. These docking results, combined with the transcriptomic findings, generate a preliminary hypothesis that requires experimental validation.

Finally, it is paramount to contextualize our *in vitro* experimental validation. Our study confirmed the significant upregulation of BAG3 and HSPB1 at both the transcriptional and translational levels in a MPP+-induced PD cell model. BAG3 is a critical co-chaperone involved in macroautophagy and the clearance of protein aggregates, which are hallmarks of PD. Its upregulation likely represents a compensatory endogenous stress response of neurons attempting to maintain protein homeostasis. Similarly, HSPB1 (HSP27) is a well-known molecular chaperone with neuroprotective properties against oxidative stress and apoptosis. By demonstrating their upregulation in response to MPP+-induced stress, we confirmed that BAG3 and HSPB1 are stress-responsive in this cellular model. Because BAG3 and HSPB1 also showed exploratory acupuncture-related temporal patterns in GSE178470, future studies should test whether acupuncture modulates these stress-response pathways using replicated longitudinal samples, acupuncture-treated experimental models, and functional perturbation experiments.

Although these findings require further empirical validation through replicated clinical cohorts, *in vitro* physical binding assays, advanced *in vivo* disease models, and molecular dynamics simulations, our integrated transcriptomic framework provides a hypothesis-generating perspective for investigating PD-associated molecular changes and their possible relationship with acupuncture intervention.

## Conclusion

The strengths of this study lie in the integration of bulk transcriptomic, single-cell transcriptomic, machine-learning, exploratory docking, and targeted *in vitro* expression analyses to prioritize PD-associated diagnostic candidate genes and generate hypotheses regarding acupuncture-related temporal expression changes. Multi-layered validation using independent cohorts and single-cell transcriptomic data further enhanced the robustness, reproducibility, and clinical relevance of our findings.

Several important limitations should be acknowledged. First, the GSE178470 RNA-seq time course was derived from one representative patient with PD who showed gait improvement after acupuncture; therefore, the Mfuzz results should be interpreted as descriptive and hypothesis-generating rather than as population-level evidence of acupuncture-related transcriptomic responses. Second, the four-gene model was trained to distinguish PD patients from healthy controls, and its performance metrics reflect diagnostic discrimination rather than prediction of acupuncture response. Third, feature screening was not fully nested within each cross-validation fold, and larger independent cohorts with longitudinal clinical outcomes are needed to validate the stability and clinical relevance of the gene panel. Finally, the single-cell, molecular docking, and MPP+-induced cellular experiments provide supportive but exploratory evidence, and functional studies are required to determine whether these candidate genes are mechanistically involved in acupuncture-related effects in PD.

Despite these limitations, our study provides a systematic integrative transcriptomic analysis that prioritizes PD-associated diagnostic candidate genes with exploratory acupuncture-related temporal patterns. By combining bulk transcriptomic screening, machine-learning-based gene prioritization, single-cell expression mapping, exploratory molecular docking, and targeted *in vitro* validation, this work offers a structured framework for linking disease-associated transcriptional changes with potential acupuncture-related molecular responses. The four-gene panel identified here may serve as a useful candidate set for future diagnostic and mechanistic studies, while the observed stress-responsive expression of BAG3 and HSPB1 provides biological support for further investigation in PD-related experimental models. Future studies incorporating replicated longitudinal acupuncture cohorts, clinical outcome measures, and functional validation will be important for determining whether these candidate genes are mechanistically involved in acupuncture-related effects in PD.

## Data Availability

Publicly available datasets were analyzed in this study. This data can be found here: GSE178470 (longitudinal transcriptomic profiles of PD patients undergoing acupuncture): https://www.ncbi.nlm.nih.gov/geo/query/acc.cgi?acc=GSE178470 GSE68719 (differential gene expression profiles comparing PD patients to healthy controls): https://www.ncbi.nlm.nih.gov/geo/query/acc.cgi?acc=GSE68719 GSE239454 (independent validation cohort): https://www.ncbi.nlm.nih.gov/geo/query/acc.cgi?acc=GSE239454 GSE157783 (single-cell RNA sequencing data from human midbrain): https://www.ncbi.nlm.nih.gov/geo/query/acc.cgi?acc=GSE157783.
